# Feasibility and efficacy of TouchCare system using application for older adults living alone: a pilot pre-experimental study

**DOI:** 10.1186/s12877-022-03482-w

**Published:** 2022-10-14

**Authors:** Jo Woon Seok, Yu-Jin Kwon, Hyangkyu Lee

**Affiliations:** 1 College of Nursing, Mo-Im Kim Research Institute, Yonsei University, 50-1, Yonsei-Ro, Seodaemun-gu, Seoul, 03722 Republic of Korea; 2 Department of Family Medicine, Yonsei University College of Medicine, Yongin Severance Hospital, Yongin, Gyeonggi-do 16995 Republic of Korea

**Keywords:** Human body communication, Life logging, Wearable device, Ambient intelligence, Context-aware artificial intelligence, Elderly, Health care

## Abstract

**Background:**

With the number of older people living alone continuously rising, health-monitoring systems using information and communication technology (ICT) have been developed to manage their health issues. Life logging and human body communication sensor, types of ICT, have been adapted to manage and monitor health status of the elderly. However, its feasibility and efficacy remain unclear. This study aimed to examine the feasibility of TouchCare system which combined life logging with human body communication technology and its effect on the physical and psychological status of older adults living alone.

**Methods:**

The TouchCare system, which consisted of a wearable watch, touchpad sensors, TouchCare application, and context-aware artificial intelligence, was developed by DNX Co. Ltd and used by the participants for 5 months. Out of the 111 selected participants, 91 replied to the satisfaction survey, and 22 participated in further investigation regarding their physical and psychological status. Finally, health assessment from 14 participants and sensor data from 13 participants (mean age = 77.4; SD = 3.8) were analyzed to compare their health status and health-related behaviors before and after use of the system.

**Results:**

Out of the 91 participants who took the survey, 51.6% were satisfied with the system. Nutritional status (pre-intervention (10.6 ± 2.0) vs. post-intervention (11.8 ± 1.9), *P* = 0.04) and fall efficacy (pre-intervention (89.2 ± 15.3) vs. post-intervention (99.9 ± 0.5), *P* = 0.001) significantly improved after use of the system. Chronic pain (pre-intervention (4.8 ± 2.5) vs. post-intervention (4.4 ± 3.7), *P* = 0.78) and depressive symptoms (pre-intervention (5.7 ± 3.9) vs. post-intervention (5.4 ± 3.1), *P* = 0.60) reduced, while cognitive function (pre-intervention (4.1 ± 1.4) vs. post-intervention (4.6 ± 1.1), *P* = 0.15) and physical performance related to walking improved (pre-intervention (3.9 ± 0.2) vs. post-intervention (4.0 ± 0), *P* = 0.35), but were not significant. Behaviors related to physical activity and gait improved after use of the system; touch counts of refrigerator and microwave also increased with a decrease in night touch counts.

**Conclusions:**

The TouchCare system was acceptable to older people living alone, and it efficiently managed their daily living while promoting their health-related behaviors. Further experimental studies are required to verify the effectiveness of the system, and to develop the system which meet the individualized needs of older people living alone.

**Supplementary Information:**

The online version contains supplementary material available at 10.1186/s12877-022-03482-w.

## Background

With the continuously expanding older population, the number of older adults living alone has been also rising [1]. Older adults living alone show worse physical, mental, and emotional health status, compared to those living with relatives [2]. Furthermore, living alone has been found to be correlated to depressive symptoms [3], resulting in lower quality of life in older adults [4]. Given that older adults living alone are prone to be frail [5], they are at increased risk of a fall that leads to hospitalization and interruption of daily life [6]. Thus, methods of early intervention should be developed and put in place for older adults living alone to manage their health status, improve their health behaviors, and maintain daily living.

Person-centered care, a perspective to encourage care receivers to be active objects of care designing care reflecting their individualized needs, preferences, and values [7]. Adaptation of person-centered care for older adults effectively improves health behaviors and health-related indicators [8]. However, since there are limited human and material resources, numerous technologies to monitor and manage health status of older adults have been recently developed and are considered to be cost-effective [9, 10]. Use of assistive technology positively influenced the well-being of older adults, as well as reduced their loneliness [11], and led to them maintaining their physical and mental health, and sustain life to age-in-place, i.e., getting older at home [12].

Sensor-based monitoring system has been employed to evaluate the health status of older adults, and recognize their emergencies [13]; however, its application in daily life has been limited due to discomfort caused in wearing or use [13], limited information monitored [14], and deficient user-friendly services for both older adults living alone and their caregivers [15]. To monitor health status more effectively and less costly, various wearable sensors which can sense one’s physiological status and motion, are developed and adopted in our daily life [16]. However, wireless technology used in the sensor such as Zigbee, Bluetooth, and Ultrawide Band (UWB) have drawbacks to apply for gaining accurate information continuously; for instance, large signal leakage can interfere with accuracy of data acquisition. To avoid these problems, human body communication technology has recently been attempted to transfer wireless information related to one’s health status directly to external device, e.g., smartphone [17].

Human body communication, a technology that transmits bodily information in the form of electricity, and can transfer parameters obtained from wearable health-monitoring devices [17, 18]. It can directly deliver the information about which things the user specific touch, whereas usual ambient sensors used for monitoring older adult’ daily living and falls consist of motion sensor which only detects one’s motion and enter in the specific space [19, 20]. However, since data from these sensors should be interpreated in special context [21], one’s activity in daily life or environmental and situational informations are also needed to determine the unhealthy gathered information through a self-monitoring application (app), overcome several disadvantages of sensors, and effectively communicate with users to motivate healtbehaviors and an emergency that poses a health hazard more accurately.

Life logging is a technology that is used to track the user’s daily life and monitor health-related behaviors, as well as motivate them [22, 23]. Recent studies have adapted life logging technology to older adults to prevent memory deficits [24] and reduce sedentary activity [25, 26]. Moreover, it has strength in gaining information about the user’s behaviors by suggesting contexts in situation judgement when combinded with sensor data [27] and can be used to provide user-oriented gathered information through a self-monitoring application (app), overcome several disadvantages of sensors, and effectively communicate with users to motivate health-related behaviors [28]. However, evidence of its effectiveness are limited; thus, it needs to be further investigated.

In this study, we developed a system that combined, for the first time to the best of our knowledge, life logging with human body communication technology (TouchCare system) and adapted it to older adults living alone.

The primary purpose of this study was to assess the feasibility of the system developed for older adults living alone. To achieve this goal, we investigated the 1) acceptability and satisfaction of the system, 2) efficacy of the system in monitoring physiological and psychological health status, and 3) effectiveness of the system on health-related behaviors. This study will support the benefits of TouchCare system to monitor health of older adults and provide a system which met older adults’ individualized needs and preferences using an app and artificial intelligence (AI).

## Material and methods

### Design of the study

This study was a pilot single group pre/post-test with mixed methods was conducted on older adults living alone over a period of 5 months. Participants who enrolled in the study were provided the TouchCare system that consisted of a touch tag sensor, wearable watch, and the TouchCare app, as well as a context-aware AI named “Suni”. A skilled researcher visited their home, installed the system, and educated them orally and with written instruction before the study began. At the end of 5 months, they were requested to participate in a telephonic or online survey that asked them about their satisfaction with the system.

Demographic information, history of disease, comorbidities, and health-related behaviors, as well as psychological and physiological health status through reliable indicators and laboratory tests before and after the use of the system were collected for the older adults who agreed to participate.

### Development of TouchCare system

**Fig. 1:**
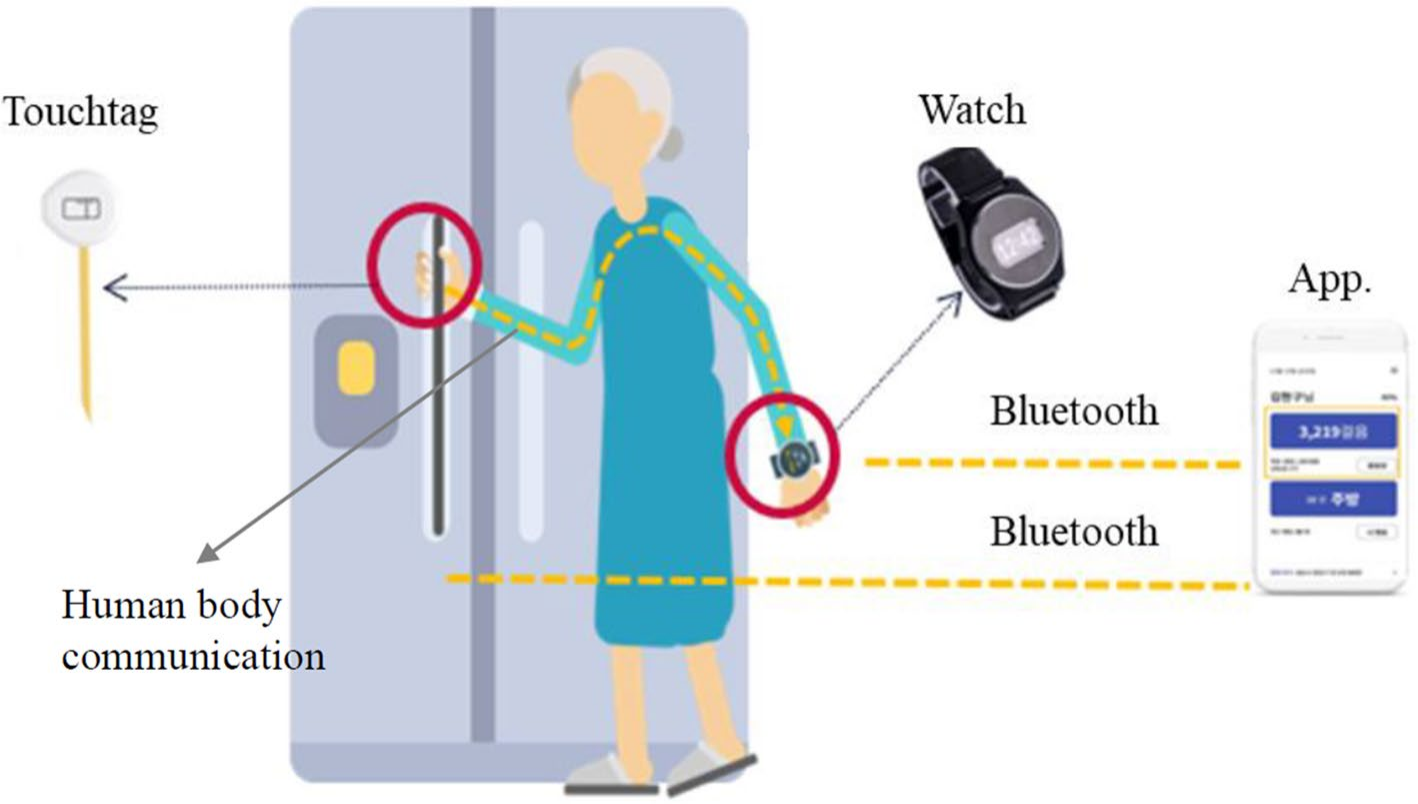
Schematic diagram of the life logging system. *Abbreviations: App* Application. Note: Touchtag is a name of sensor and watch is used to gather individualized information of using the items attaching touchtag

Automatic collection of “life log” data was facilitated by the TouchCare system developed by DNX Co. Ltd (Kyungki-do, South Korea). The system registered an entry each time a user physically touched items in their environment, and also measured indicators related to physical activity such as daily step-count, stride length, and gait. It consisted of the following key components: (1) ‘touchtags’, sensors attached to key household items such as refrigerator, microwave, sink, toilet, TV remote, etc. (2) A wearable smart watch that detected movement, posture, step-count, etc. (3) A smartphone app that received data from both, the touchtags and wearable watch. (4) Context-aware AI voice messaging via the app that delivered interventions upon detecting sub-optimal behavior, potential issues, etc. The key components of the system and principle of delivering data are shown in (Fig. 1).

#### Touchtags and wearable watch

Data received from the touchtags and wearable watch were analyzed both on-device and in the cloud to build behavior profiles of a user, which were subsequently used to determine required interventions.

#### Touchcare application

The smartphone app called TouchCare had versions for care-receivers that showed key indicators such as step-count and were used to deliver voice messaging. The version for caregivers highlighted behavior anomalies of those under their care, showed a live motion graph, and sent emergency alerts. In addition, the app also provided various inteventions which ranged from general well-being promotion in the form of singing activities, storytelling, and quizzes to behavioral change prompts for identified issues such as excessive TV consumption, lack of physical activity, and poor eating habits.

#### Context-Aware Artificial Intelligence (AI)

The virtual care dialog flows were produced through large-scale modular behavior networks with inferred contexts. They were designed to generate suitable messages to trigger user’s positive behavioral change, decrease loneliness, and promote safe activity in order to minimize accidents. The context-aware AI system consisted of four component modules: data perception, context recognition, emotion treatment, and dialog management. The perception module gathered information about the user from the touchtags and smart watch. The user context recognition module inferred high-level contexts about the user. A probabilistic model was used to deal with the complex environment and the uncertainties of the information sources. The emotion treatment module produced an emotional state of the virtual caregiver “Suni” using Russel’s arousal-variance model. The dialog module finally generated timely personalized suitable dialog messages. The message delivered to user via voice in touchcare app, regardless of whether the app in smartphone was on or off. The interventions to promote healthy behavior and prevent cognitive decline or depressive symptoms were provided via touchcare app based on the context determined by various data, e.g., sedentary actitivies, moment touching the objects in the house, times of outings, which supported by context-aware AI system.

### Participants and procedure

**Fig. 2:**
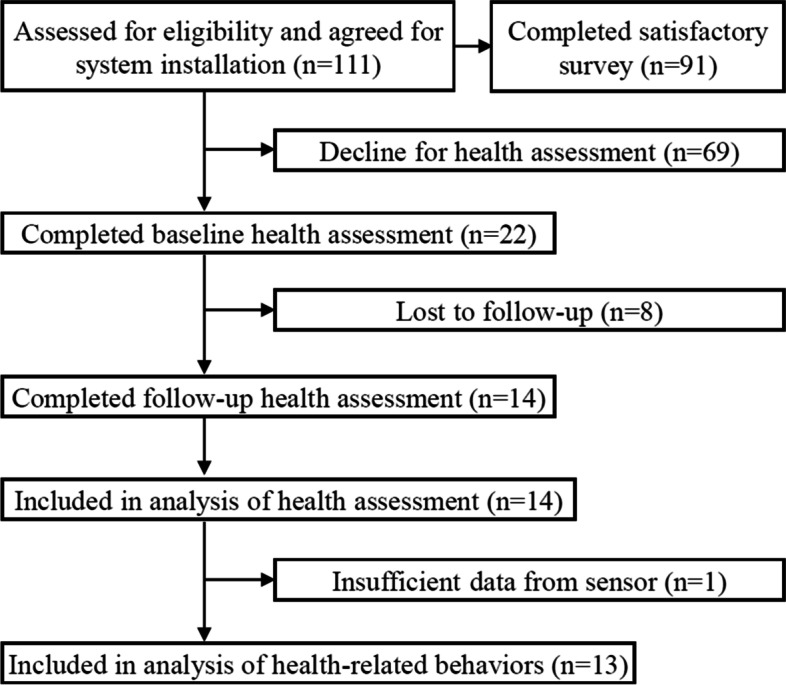
Flow chart of participants

Older adults living alone were recruited through a senior welfare center located in Yongin-si, Gyeonggi-do, Republic of Korea from October 31,2019 to January 28,2021, and screened for eligibility by a researcher. Inclusion criteria for participation were as follows: (1) age over 65 years, (2) living alone, (3) able to use smartphone. A total of 111 older adults living alone applied for the study, and those who understood the contents of study and agreed with the terms of further investigation were enrolled once they provided written informed consent. The system was installed at the homes of the participants; out of the 111, 91 participants completed the questionnaires about feasibility and satisfaction of the system after using the system for 5 months. Twenty-two participants visited the Yongin Severance Health Check-up Center for the health examination. Demographics, history of disease, and baseline health status were assessed by trained research staff and a clinical medical staff member before installing TouchCare system. Out of 22, 8 participants refused to engage in the follow-up health examination. Finally, data from 14 participants were used for further analysis. However, due to insufficient records from sensors of a participant, we included 13 particpants in the analysis of health-related behaviors. The flow chart of participants is provided in Fig. 2.

### Measures

#### Users’ satisfaction with TouchCare system

To investigate the user satisfaction with TouchCare system, we conducted a survey via phone call or online with 91 older adults living alone who experienced the system for 5 months. The questionnaire used consisted of 5 parts: “Management of system,” “Improvement in quality of care,” “Increasing emotional stability,” “Reduction in loneliness,” and “Overall.” Scores for each part were measured using a 5-point Likert scale. In addition, older adults participating in the survey also provided qualitative feedback about the use of the system.

#### Demographics and background information

The background and demographic information collected included sociodemographic status (age, gender, marital status, occupational status, and educational level), past and present medical history (past and family history of disease, comorbidities, and medication), and healthy behaviors (smoking, alcohol consumption, and exercise).

#### Geriatric assessment

To examine physical and psychological function of older adults, we assessed nutritional status, depressive symptoms, pain, frailty, fall’s efficacy, cognition status, and physical performance related to walking where these included in Korean version of comprehensive geriatric assessment. Korean version of geriatric assessemnt, developed by korean society of family medicine, consisted of various domains including basic medical asseessment (senses, pain, health promotion behavior, and voiding-related problem), psychological and cognitive function (cognition, delirium, and depression), frailty (physical function and fall), social support, and considerations on the death. Since this version was developed to select health problems from older adults and to provide individualized appropriate care to them, the tool included multiple valid tools which fit to assess each domains based on previous study [29, 30]. In this study, we assessed physical and psychological function of older adults using some of the tools included in comprehensive geriatric assessement, e.g., nutritional status, cognitive function, pain, depressive symptoms, frailty, fall’s efficacy and physical status, due to the limited time required for evaluation and coordination of multidisciplinary specialties. Baseline assessment was conducted before installing the system, and a follow-up assessment took place 5 months after the system had been in use.

##### Nutritional status

The Mini Nutritional Assessment (MNA) is a simple, noninvasive, well-validated screening tool for early detection of malnutrition in elderly [31]. In this study, we used the short form of the MNA, consisting of six questions on food intake, weight loss, mobility, psychological stress, or acute disease, the presence of dementia or depression, and body mass index. The maximum score for this part is equal to 14. A score equal to or higher than 12 indicates that the subject under study has an acceptable nutritional, a score <12 indicates that the subjects under malnutrition risk [32]. Nutritional status was screened with the Korean version of the Mini-Nutritional Assessment (MNA), in which lesser scores indicate higher risks for malnutrition [33].

##### Numerical rating scale for pain

Older persons are likely to suffer both acute and chronic pain [34]. Pain in older persons are associated with increased risk of depression, sleep disturbance, and impaired physical function [35]. Numeric rating scale (NRS) for pain intensity are preferred by elderly. NRS for pain are asked the patient to rate their pain from 0 to 10, with 0 representing one end of the pain continuum (e.g., no pain) and 10 representing the other extreme of pain intensity [35].

##### Depressive symptoms

Depressive symptoms were assessed using the Korean versions of the Short Geriatric Depression Scale (SGDS) developed by Bae et al. The SGDS is widely used for screening depression of older adults. It consists of self-reported 15 items including “Are you satisfied with your life?”, “Have you recently reduced your activities or interests?”, and each item is scored based on yes-or-no responses. Total possible scores range from 0 to 15, and higher scores indicate showing more depressive symptoms. The SGDS was validated for use in Korean elderly through both clinical practice and research, and a score of 8 was suggested as the optimal cut off point to screen for depressive disorders [36].

##### Frailty

Frailty was screened using the Korean Frailty Index (KFI) developed in 2010 by a consensus panel of the Korean Geriatrics Society [37]. This indicator consisted of 8 items including a history of hospitalization, subjective sense of healthiness, polypharmacy including Korean herbal medicines, subjective weight loss in the previous month, questions on depression and incontinence, the timed up and go test, and a question regarding visual and hearing impairments and was validated for the older Korean adults (Cronbach’s α=0.65) [38]. The total scores of the KFI range from 0 to 8. A score equal to higher than 5 indicated frailty. A score 3-4 indicated for prefrailty.

##### Fall’s efficacy

The Korean version of the Fall efficacy scale (FES) was used to evaluate confidence to perform daily activities without loss of balance and falls. The FES consists of 10 items: 1. Taking a bath or shower, 2. Reaching into the closet, 3. Doing light housekeeping, 4. Walking around the house, 5. Getting in and out of bed, 6. Getting up at night to go to the bathroom, 7. Getting dressed and undressed, 9. Doing personal hygiene, and 10. Using the toilet. Scores range from 1 (cannot do it at all) to 10 (can do it perfectly). The total score for evaluating fall efficacy is the sum of each of 10 items. Higher scores indicate having lower anxiety with fall and a score <80 indicated that high risk of fall [39, 40].

##### Cognitive function

Cognitive function was evaluated using the Korean version of mini-cog, a 3-minute brief and simple cognitive screening questionnaire for early detection of cognitive impairment in older adults [41]. It includes 3-item recall, and clock drawing. 2 points for a normal clock or 0 points for an abnormal clock drawing. Total score are ranges from 0 to 5. A score equal to higher 3 indicated lower risk of dementia.

##### Physical status

The Short Physical Performance Battery (SPPB) was well-established instrument for the measurement of physical performance in older adults [42]. The standing balance tests included tandem, semi-tandem and side-by-side standing, and the participants were timed until they moved or 10 s had elapsed. To assess gait speed, the participants were asked to walk 4 m at their regular pace. We conducted two SPPB (balance test and gait speed). The SPPB was scored from 0 to 4, with higher scores reflecting better function [43].

#### Anthropometrics and laboratory tests

Height and weight were measured to the nearest 0.1 cm and 0.1 kg, respectively. Body mass index (BMI) was calculated as body weight divided by height squared (kg/m2). Waist circumference (cm) was measured in the horizontal plane midway between the lowest rib and the iliac crest. Calf circumference was measured at the calf’s greatest girth using an inelastic tape measure with the participant in an upright position. Systolic blood pressure and diastolic blood pressure were defined as the average of the two measured values. Blood samples were collected after a minimum of 8 hours of fasting. Fasting glucose, insulin, total cholesterol, triglyceride, high-density lipoprotein cholesterol, and low-density lipoprotein cholesterol were measured. Body composition data were collected using whole-body Dual energy X-ray analysis (DEXA) (Horizon DXA System; Hologic Inc., Bedford, MA, USA).

### Data extraction and analysis

#### Sensor data from TouchCare system

##### Data extraction

To measure physical activity levels of the participants, numerous data were collected from the touchtag sensors and wearable watch. Overall activity levels within the participant’s home environment were collected by recording the quantity and times of touch events as follows: (1) Each time a household item containing a touchtag was touched the system captured a) the item identifier and b) the timestamp. Algorithms merged multiple and continuous touches into a single ‘touch event’ – for instance, if the TV remote was touched multiple times within one minute it would be registered as a single “touch event.” (2) The watch had a gyro and accelerometer sensor which sent 6-axis data when the caregiver wanted to see the live motion graph or an internally designed flow happened. Emergency alert event data was sent to the caregiver. (3) To capture outings, the smartphone sent GPS information regularly, and motion data from the watch’s gyro sensor was sent for context recognition such as waking or walking.

##### Data analysis

Sensor data recorded from September, 2020 to January, 2021, was used for further analysis.

Activity counts, meaning total step counts when participants wearing smartwatch. When the sensor detected stepping, total stepping times and its length were also counted.

Mean activity counts per minute were calculated from the total activity accounts divided by total valid times, based on a previous study [44]. Ratio of low activity counts, defined as rates of sedentary activitives , were caculated from dividing the number when activity counts below 40 by total number when acitivity counts were recorded. Gait speed and length were calculated from the total length and time of a walk.

Touch counts of the sensor attached to the refrigerator and microwave were used to indirectly represent how many attempts were made toward eating and cooking. Total touch counts were divided by total valid days, to remove bias related to favorability for use of the system. Touch counts recorded between 9:00 pm and 5:00 am were counted as night touch [45].

Similarly, days of outing were counted by dividing all counts of outing by the total valid days. All the sensor data were arranged by month and calculated average counts with deviation.

#### Statistical analysis

Statistical analysis was conducted using R 4.0.2 (R Foundation for Statistical Computing, Vienna). Data from geriatric assessment, anthropometrics, and laboratory tests were tested for normality using the Shapiro-wilk test. Data were presented as mean ±standard deviation (SD) or number (%). Then, significance of difference between scores evaluated before and after use of the system was tested using a paired T-test with normally distributed data, and using Wilcoxon signed rank test with the data not normally distributed.

## Results

### Feasibility and acceptability of using touchcare system

To examine whether older adults were satisfied with TouchCare system and found it accessible, we assessed satisfaction categorized by 4 items. Out of the 111 older adults who experienced TouchCare system, 91 participated in the survey regarding satisfaction with the system. In detail, 47 participants were very satisfied and 29 participants were satisfied with the system, where 15 participants were neither satisfied nor dissatisfied. Totally, over half of the older adults were very satisfied with the system generally and with its management. Furthermore, they found that it improved the quality of care and reduced their loneliness, as well as increased their emotional stability. These results suggested that TouchCare system was effectively managed and it acted as a good care manager as it supported older adults with their psychosocial status (Table 1).

**Table 1:** Satisfaction for using TouchCare system (*N* = 91)

Measures	Participants response, n (%)					Average points, Mean ± SD
	Very satisfied (5-point)	Satisfied (4-point)	Neither satisfied nor dissatisfied (3-point)	Dissatisfied (2-point)	Very dissatisfied (1-point)	
Management of system	48 (52.7)	24 (26.4)	17 (18.7)	2 (2.2)	0 (0)	4.2 ± 0.72
Improvement in quality of care	49 (53.8)	26 (28.6)	15 (16.5)	1 (1.1)	0 (0)	4.2 ± 0.66
Increasing emotional stability	42 (46.2)	33 (36.3)	15 (16.5)	0 (0)	0 (0)	4.1 ± 0.63
Reduction of loneliness	46 (50.5)	32 (35.2)	13 (14.3)	0 (0)	0 (0)	4.2 ± 0.54
Overall	47 (51.6)	29 (31.9)	15 (16.5)	0 (0)	0 (0)	4.2 ± 0.58

In addition, at the end of the survey, participants mentioned that they became more active, had better quality of life, and interacted frequently with an intelligent AI caregiver called Suni.

“I’m a big fan of Suni. I love you, Suni.” [Participant (P)1]

“Suni warns me when I watch TV too late. So, I try to go to bed early.” (P2)

“Because Suni told some old stories, I was not bored.” (P3)

“COVID-19 made me lonely, but it was good to have Suni by my side.” (P4)

However, some older adults thought that this system was not helpful and it was inconvenient for use in daily life.

“Contained services are fun and good, but I don’t think it’s necessary.” (P5)

“It’s uncomfortable to use smartwatch.” (P6)

### Demographic characteristics of participants

Information about sociodemographic status, past and present medical history, and healthy behaviors has been summarized and presented in Table 2. The mean age of participants was 77.3 ± 3.8 years.

**Table 2:** Sociodemographic and background information of the participants (*N* = 14)

	Variables	Mean (SD) or N (%)
**Sociodemographic information**		
Age		77.3 (3.8)^a^
Sex	Male	3 (21.4)
	Female	11 (78.6)
Marriage	Married	1 (7.1)
	Bereaved or divorced	13 (92.9)
Have Job	Yes	2 (14.3)
	No	12 (85.7)
Education	High school or below	14 (100)
	College or above	0 (0)
**Comorbidities**	Cancer	2 (14.3)
	Hypertension	10 (71.4)
	Diabetes Mellitus	3 (21.4)
	Dyslipidemia	4 (28.6)
	Myocardial infarction or Angina	1 (7.1)
	Cerebral infarction	2 (14.3)
**Family history**	Diabetes Mellitus	4 (28.6)
	Myocardial infarction or Angina	1 (7.1)
	Cancer	5 (35.7)
**Smoking**	Yes	2 (14.3)
	No	12 (85.7)
**Regular alcohol drinking**	Yes	3 (21.4)
	No	11 (78.6)
**Regular exercise**	Yes	1 (7.1)
	No	13 (92.9)

^a^Data indicates mean value with standard deviation (SD)

Participants consisted of 78.6% females, and 92.9% of them were bereaved and did not have a job (85.7%). The educational level of all participants was high school level or below. They suffered from various chronic diseases: 2 of them had cancer, 10 had hypertension, 3 had diabetes, 4 had dyslipidemia, and 3 had myocardial or cerebral infarction. The proportions of the participants who regularly exercised, smoked, or drank alcohol were 7.1%, 14.3%, and 21.4%, respectively. The data reflected that the participants had specialized needs for continuous health monitoring and promotion of physical activity.

### Changes in functional ability, physical health, cognition, and mental health before and after use of touchcare system

We compared the functional ability, physical health, cognition, and mental health of older adults via geriatric assessment before and after use of TouchCare system. As the results presented in Table 3 indicate, the mean values of nutritional status improved significantly after using the system, compared to baseline assessment (pre-intervention; mean (SD), 11.8 (1.9) vs post-intervention; mean (SD), 10.6 (2.0), *P*=0.04). In addition, participants showed significantly high FES variation (pre-intervention; mean [SD], 99.5 (0.5) vs post-intervention; mean (SD), 89.2 (15.3), *P*=0.001). Frailty score lowered after intervention; however, there was no statistical significance (pre-intervention; mean (SD), 3.3 (2.1) vs post-intervention; mean (SD), 3.0 (2.0), *P*=0.6)./ 3.0 SD=2.0; *P*=0.6). Depressive symptoms and chronic pain decreased after 5 months of use of the system (pre-intervention; mean (SD), 5.7 (3.9) vs post-intervention; mean (SD), 5.4(4.1), *P*=0.6; pre-intervention 4.8 SD=2.5; post-intervention 4.4 SD=3.7; *P*=0.78), but were not statistically significant. Although there was no statistically significant change, cognitive function and physical function of lower limb also improved, suggesting long-term use of the system could effectively manage psychological and physical health of older adults living alone.

**Table 3:** Changes before and after use of TouchCare system (*N* = 14)

Measures	Pre-intervention^a^	Post-intervention^a^	*P*
MNA (nutrition)	10.6 (2.0)	11.8 (1.9)	0.04‡
NRS (pain)	4.8 (2.5)	4.4 (3.7)	0.78
SGDS (depression)	5.7 (3.9)	5.4 (4.1)	0.60
FES (fall)	89.2 (15.3)	99.9 (0.5)	0.001§
Frailty index (frailty)	3.3 (2.1)	3.0 (2.0)	0.60
Mini-Cog (cognition)	4.1 (1.4)	4.6 (1.1)	0.15
SPPB-balance test (physical function)	3.9 (0.2)	4 (0)	0.35
SPPB-gait speed (physical function)	1.9 (0.3)	2 (0)	0.15

*Abbreviations: MNA* Mini-Nutritional Assessment, *NRS* Numeric Rating Scale, *SGDS* Short Geriatric Depression Scale, *FES* Fall Efficacy Scale, *SPPB* Short Physical Performance Battery

^a^Data represents average value with standard deviation

^‡^*P* value < 0.05, §*P* value < 0.01

Table 4 shows the changes in body composition and laboratory tests before and after system use. We found favorable changes in body weight, calf circumference, metabolic rate, and abdominal fat mass. However, there was no statistical significance.

**Table 4:** Changes before and after use of TouchCare system (*N* = 14)

Measures	Pre-intervention^a^	Post-intervention^a^	*P*
Weight (kg)	55.0 (12.3)	55.8 (10.7)	0.96
Waist circumference (cm)	77.5 (24.4)	79.3 (8.3)	0.32
BMI (kg/m^2^)	24.2 (4.9)	24.5 (4.7)	0.11
Calf circumference (cm)	30.8 (9.5)	32.6 (3.1)	0.79
Systolic blood pressure (mmHg)	131.9 (40.7)	140.0 (16.5)	0.76
Diastolic blood pressure (mmHg)	72.1 (23.4)	72.6 (13.8)	0.26
Fasting glucose (mg/dl)	108.6 (16.0)	119.4 (64.7)	0.62
Insulin (IU/L)	7.4 (5.4)	8.6 (8.9)	0.89
HOMA-IR (mg/IU)	1.9 (1.4)	2.7 (3.0)	0.83
Hemoglobin (mg/dl)	12.9 (1.4)	13.0 (1.3)	0.71
Triglyceride (mg/dl)	108.4 (65.3)	108.3 (49.7)	0.99
Cholesterol, total (mg/dl)	175.9 (45.1)	162.6 (28.1)	0.53
HDL-C (mg/dl)	61.0 (17.7)	54.4 (20.5)	0.56
LDL-C (mg/dl)	93.2 (40.3)	83.1 (27.3)	0.67
Creatinine (mg/dl)	0.9 (0.4)	0.9 (0.3)	0.20
AST	23.0 (4.7)	24.1 (8.3)	0.81
ALT	16.7 (9.7)	17.6 (9.3)	0.76
Limb BMC (g)	721.2 (187.4)	730.3 (195.3)	0.87
Limb fat (g)	8458.9 (3507.0)	8567.7 (3505.7)	0.45
Limb lean mass (g)	12,628.4 (2584.0)	12,575.2 (2416.0)	0.60
Muscle mass (kg)	19.9 (3.9)	20.5 (3.7)	0.28
Fat mass (kg)	19.5 (9.3)	20.5 (3.7)	0.94
Body fat ratio (%)	19.5 (9.3)	20.0 (7.5)	0.90
Waist-hip ratio	34.2 (11.9)	35.9 (9.3)	0.96
Abdominal fat (cm^2^)	153.1 (90.0)	149.4 (89.1)	0.84
Subcutaneous fat (kg)	2.9 (1.7)	3.1 (1.4)	0.84
Basal metabolic rate(kcal)	1065.2 (314.6)	1157.9 (145.66)	0.61
Daily required energy (kcal)	1748.7 (233.1)	1783.0 (225.3)	0.32

*Abbreviations: BMI* Body Mass Index, *HOMA-IR* Homeostatic Model Assessment for Insulin Resistance, *HDL-C* High-Density Lipoprotein Cholesterol, *LDL-C* Low-Density Lipoprotein Cholesterol, *AST* Aspartate aminotransferase, *ALT* Alanine transaminase, *BMC* Bone Mineral Contents

^a^Data represents average value with standard deviation

### Alteration in health-related behaviors

**Fig. 3:**
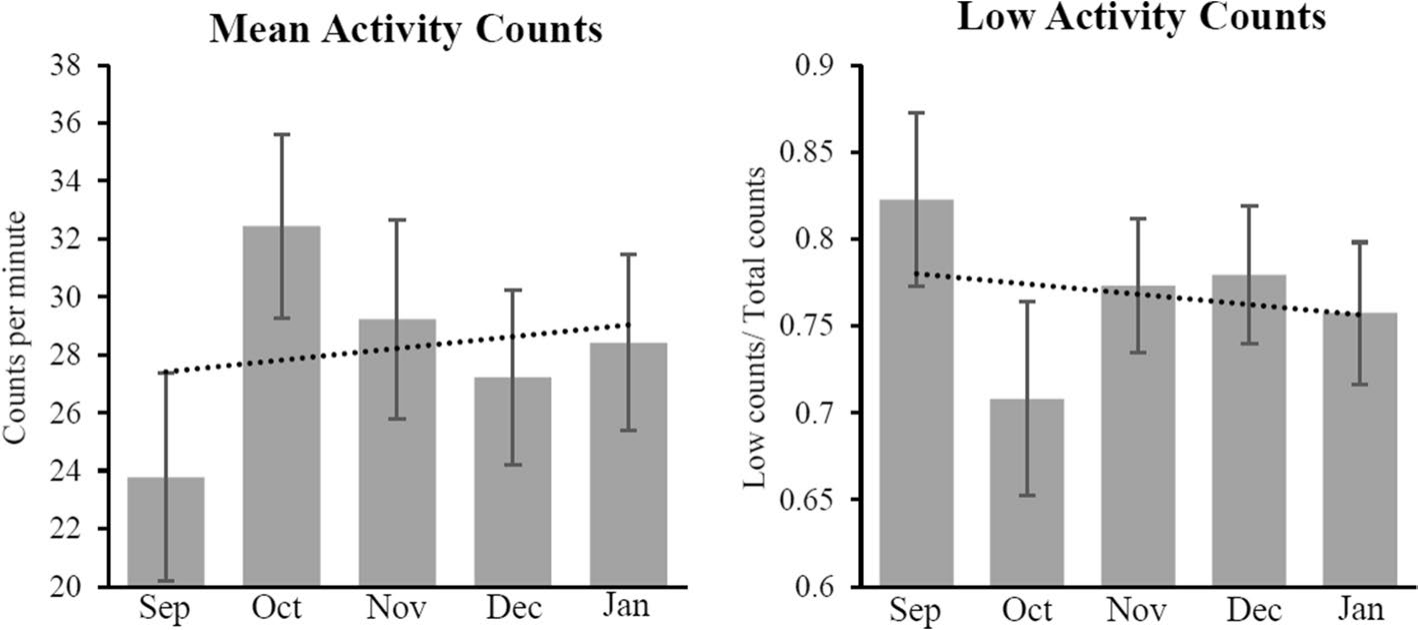
Trend of older adults’ activity counts after use of the system. *Note:* Data represents mean value with standard error (SE) in the indicated month. Dot line indicates trend line of change in activities. *Abbreviation: Sep* September, *Oct* October, *Nov* November, *Dec* December, *Jan* January

**Fig. 4:**
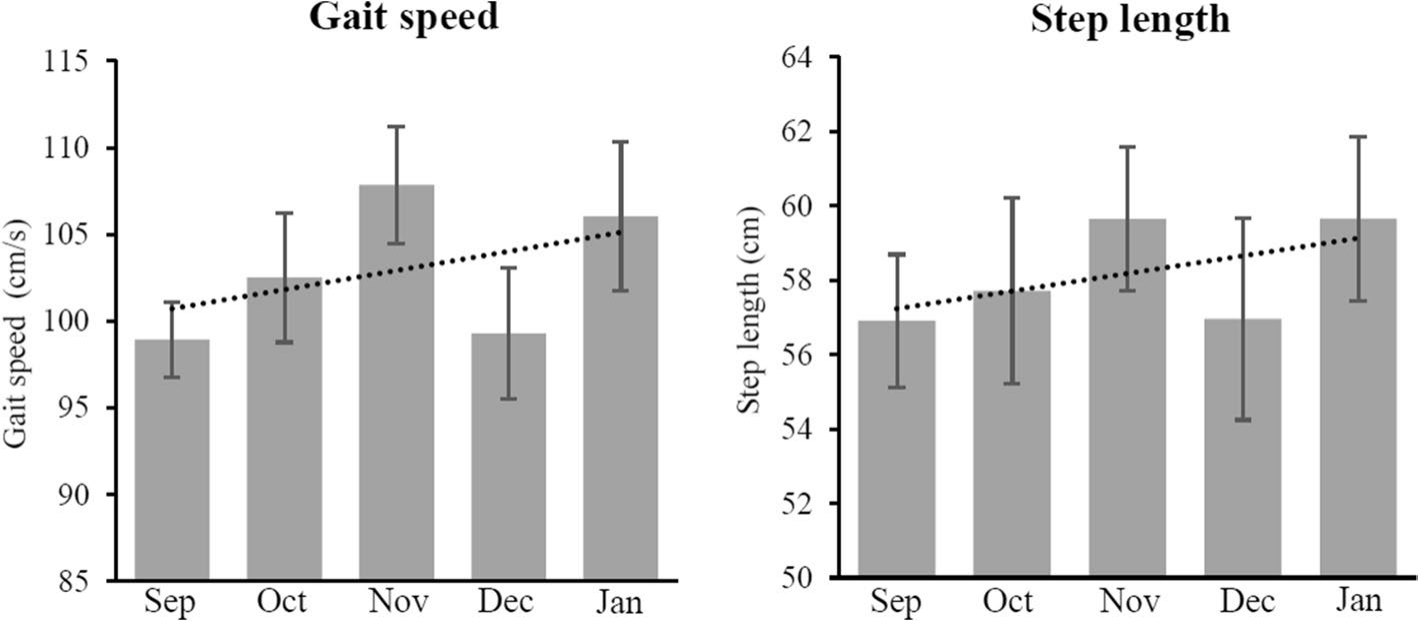
Trend of older adults’ gait after use of the system. *Note:* Data represents mean value with standard error (SE) in the indicated month. Dot line indicates trend line of change in activities. *Abbreviation: Sep* September, *Oct* October, *Nov* November, *Dec* December, *Jan* January

To investigate behavioral change before and after use of the system, we obtained data, such as walking, touch counts, and number of outings, from the sensors and app. Interestingly, mean activity counts increased a month after intervention, and then lightly decreased, but maintained higher values than beginning of the use, while the ratio of low activity was decreased and maintained in the course of use (Fig. 3). Consistently, step length and gait speed were also enhanced, compared to basal level measured in September, 2020 (Fig. 4). The data suggests that using the system could be beneficial for enhancing walking, resulting in an improvement in gait.

**Fig. 5:**
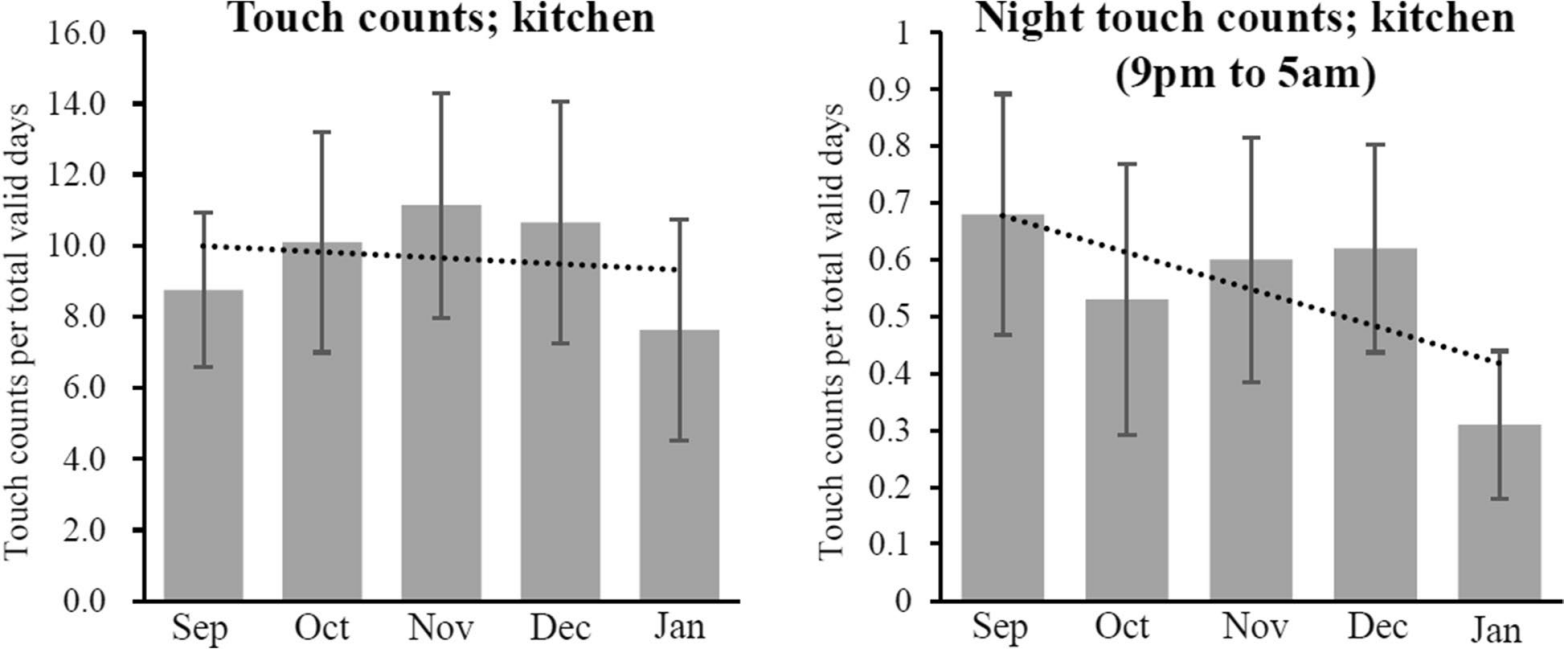
Trend of touch counts in kitchen after use of the system. *Note:* Data represents mean value with standard error (SE) in the indicated month. Dot line indicates trend line of change in activities. *Abbreviation: Sep* September, *Oct* October, *Nov* November, *Dec* December, *Jan* January

Next, we examined how patterns of activities implying eating or cooking changed dependent on time. Night eating habits were closely associated with metabolic syndrome, especially with dyslipidemia [46]. Since most participants had diabetes, hypertension, and dyslipidemia, we looked into counts of night eating habits assuming that touch counts of refrigerator and microwave closely related with activities of eating or cooking. Although all time touch counts of refrigerator and microwave were nearly unchanged after implementation of the system (Fig. 5, left), counts of night eating habits decreased with time (Fig. 5, right). The data implies that eating habits of participants changed to the healthier pattern, resulting in better nutritional status.

**Fig. 6:**
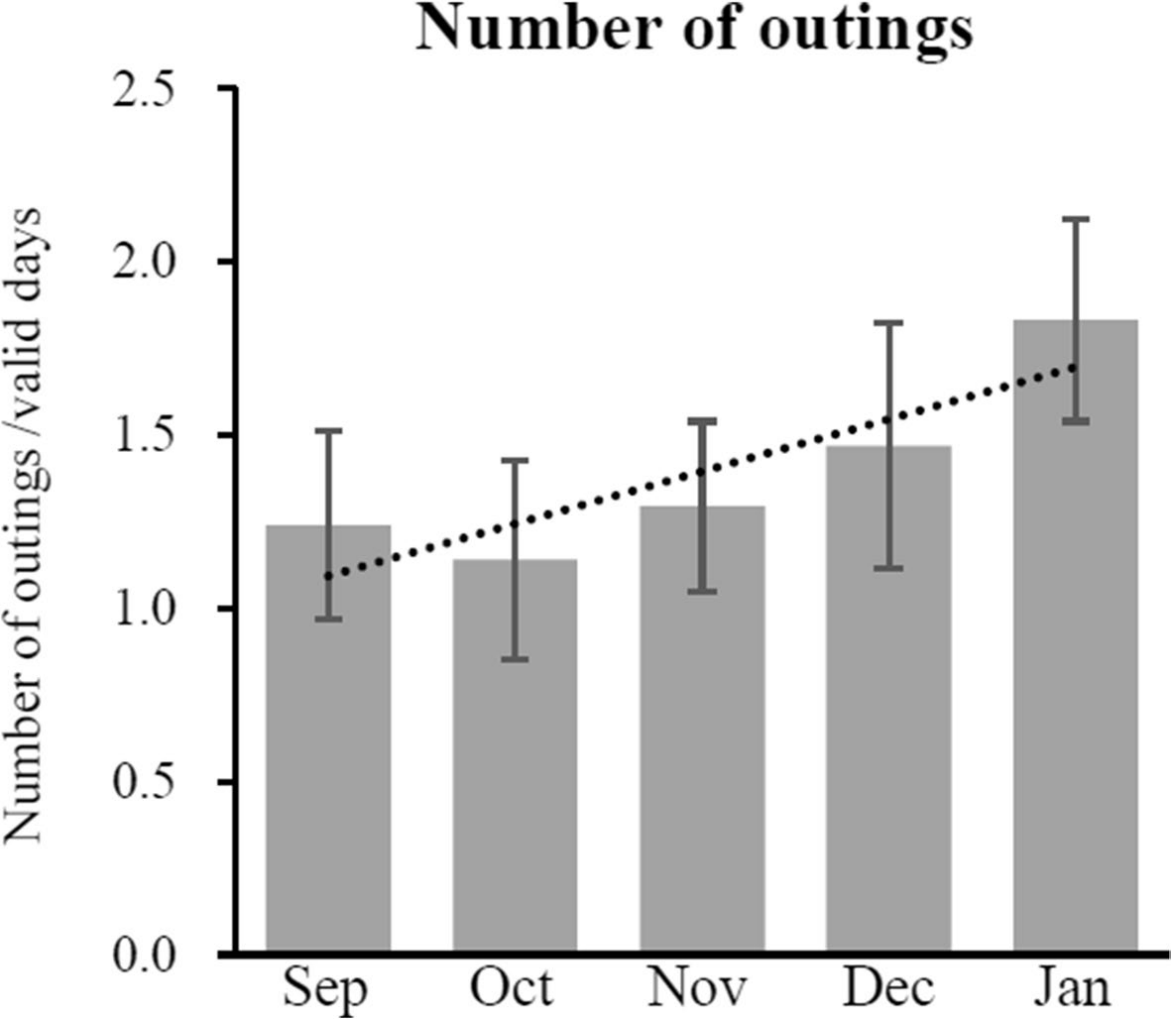
Trend of number of outings use of the system. *Note:* Data represents mean value with standard error (SE) in the indicated month. Dot line indicates trend line of change in activities. *Abbreviation: Sep* September, *Oct* October, *Nov* November, *Dec* December, *Jan* January

Finally, increased number of outings that were assumed to be related to reduction of depressive behavior appeared after the use of the system (Fig. 6).

To test whether there change in activities, touch counts, gait, and outing between those who completed the follow-up health examination and thouse who did not complete it, we also investigate patterns of their behavioral change in the full sample (*N*=22). Taken together, these data suggested that older adults became more active, improved their gait, tended to avoid night eating, and showed increased outings after use of TouchCare system.

## Discussion

This study adapted TouchCare system for older adults living alone and evaluated its applicability and effectiveness in various domains of health.

Life logging could be a beneficial tool for monitoring individualized and contextual information about activity patterns [47]; however, since one’s personal information has to be collected and preserved, it should be applicated delicately depending upon its users. Our TouchCare system was devised and developed to cater to older adults, and it provided a user-friendly app and AI with major functions to meet older adults’ specialized needs and manage their health problems and safety issues. Most of the users were very satisfied with the system and improved their health status and health-related behaviors. It is notable that the use of the system improved participants’ nutritional status and anxiety related to fall significantly, although only a small number of older adults participated in the health assessment.

Previous studies that have monitored older adults using life logging through a wearable camera also reported that life logging technology was acceptable to older adults [24, 26, 48]. Systems using life logging supported user’s memory recall and could be a successful tool for reducing cognitive defects in older adults [26, 48]. However, these studies also pointed out that the further research should consider ethical issues related to user’s privacy and develop algorithms to capture meaningful images from the records. In our study, we used sensors to detect environmental information, instead of a camera worn by the older adult; Hence, there was little possibility of exposing one’s face or behaviors directly to others, thus reducing privacy issues related to life logging systems. In addition to just monitoring the user’s behaviors, we also combined the life logging system with a user-friendly app and AI speaker that could provide detailed feedback about how to use the devices and provide health-related counseling to older adults. Furthermore, to our knowledge this is the first study to show effects of the life logging system on health status and health-related behaviors of older adults living alone.

Although regular physical activity has been reported to be important for enhancing mental health, emotional, psychological, social well-being, and cognitive function in older adults, over 80% of older adults worldwide report inactivity [49, 50]. This suggests that they should be continuously motivated to exercise or walk. This study revealed that the system effectively promoted older adults to walk and go out, resulting in improved gait and reduced depressive symptoms. Though low temperature negatively influences on physical activities in older adults, our study also showed favorable increasing in step counts and outings in the the winter season [51].

Frailty, a state of increased vulnerability related to older adults’ physical and cognitive health, results in dramatic changes in functional ability [52]. To protect older adults from irreversible damage, exercise and nutritional interventions have been proposed by previous research [52–54]. Our study found out that TouchCare system effectively improved participants nutritional status and promoted their activities, e.g. stepping, outing, although frailty was not improved significantly. However, the participants maintained the same level of frailty even after 5 months, suggesting that the system was possibly concerned with reducing vulnerability related to frailty. We proposed that use of the system could partially act as a virtual manager for maintaining physical, cognitive, and psychosocial health of older adults living alone. Furthermore, the contents related to muscle strength training and protein supplementation should be developed and delivered to them in order to improve frailty successfully [55].

In previous studies, physical and social inactivity were associated with depressive symptoms in older adults living alone in Korean populations [56, 57]. Our findings indicated that the system motivated older adults to spend time outside and increase physical activity, resulting in decreased depressive feelings. In addition, AI Suni interacted with them instead of a human which indicates that AI could be a good alternative for enhancing social interaction of older adults living alone as a large number of social robotz or AI are being developed [58, 59].

However, methods to evaluate quantity and quality of conversation through AI should be developed to show effects of context-aware AI in promoting social interaction.

Social isolation and feelings of loneliness have been reported to be critical risk factors for dementia [60, 61] which indicates that older adults living alone are at higher risk of developing cognitive defects; thus, there is a need to continuously manage their feelings, as well as cognitive status. Previous studies have also noticed that the life use of logging was acceptable to older adults with mild cognitive infarction (MCI) [26]. In our study, the participants with normal cognitive function were enrolled to verify the applicability of the technology to older adults. Thus, we could not measure significant increase in the participants’ cognitive function. However, further research on older adults with MCI or mild dementia must be conducted to measure their cognitive status using more precise instruments.

Several limitations about this study should be noted. First, the design of the study was not randomized controlled and a relatively small number of participants were enrolled; the effects of the system should therefore be explained with caution. Second, some participants who had enrolled in the physical examination, dropped out in the follow-up physical examination. As a result, the results of pre-post health status might be distorted and should be interpretated carefully. However, to overcome this error, we also tested whether completers (thouse who completed follow-up physical examination) and remainders (thouse who did not follow-up physical examination) showed differences in basal health status, and found out that there’s no significant change between two groups. (supplementary table S1- 2). Third, social distancing measures were implemented due to COVID-19 while the study was active, so it was difficult for older adults living alone to go outside. Thus, we were not able to accurately measure the effect of the system. Fourth, due to limited the time required for evaluation and need for coordination of multidisciplinary specialties, we could not perform the full comprehensive geriatric assessment. In the further study, more comprehensive geriatric assessment are needed. Finally, the quantity of sensor data that could be extracted from the touchtags was heterogeneous as it depended on the user’s preference and how participants used the system (e.g. regularity, preference, etc.).

## Conclusions

In this study, we developed a new type of health- and behavior- monitoring system named TouchCare for older adults and showed the response of its users and its effectiveness on users’ health status. Older adults living alone provided positive qualitative feedback for the system with a high rate of satisfaction. In addition, the use of the system effectively encouraged them to change their lifestyle to become healthy and increase physical and social activity. Thus, the system could act as a cost-effective virtual caregiver for older adults. Furthermore, this study will provide healthcare providers and related industry workers with evidence about efficacy of life logging when it applies to health monitoring devices. This will also help older adults to participate in using ICT and understand its effects on managing their own health better. However, further research on a larger scale is required to show the effects of the system on physical, psychological, and cognitive function.

## Supplementary Information

**Additional file 1: Supplementary table S1.** Comparing the geriatric assessment between completers and remainders. **Supplementary table S2.** Comparing the body composition and laboratory tests between completers and remainders.

## Data Availability

The datasets generated and analysed during the current study are available from the corresponding author on reasonable request.

## Abbreviations

AI: Artificial Intelligence
COVID-19: Coronavirus Disease 2019
FES: Fall Efficacy Scale
MNA: Mini-Nutritional Assessment
SGDS: Short Geriartric Depression Scale
SPPB: Short Physical Performance Battery
