# Supplementary material for: Feasibility and efficacy of TouchCare system using application for older adults living alone: a pilot pre-experimental study
Source: BMC Geriatr. 2022 Oct 14;22:799. doi: 10.1186/s12877-022-03482-w (PMC9829959; doi:10.1186/s12877-022-03482-w)
Supplement: Supplementary file 1 — Additional file 1: Supplementary table S1. Comparing the geriatric assessment between completers and remainders. Supplementary table S2. Comparing the body composition and laboratory tests between completers and remainders. [file 12877_2022_3482_MOESM1_ESM.pdf]

# Supplementary information

Supplementary table S1. Comparing the geriatric assessment between completers and remainders.

| Variables | Completers | Remainders | *P* |
|---|---|---|---|
| MNA (nutrition) | 10.6 (2.0) | 12.3 (2.1) | 0.09 |
| NRS (pain) | 4.8 (2.5) | 4.9 (2.7) | 0.91 |
| SGDS (depression) | 5.7 (3.9) | 4.3 (1.7) | 0.23 |
| FES (fall) | 89.2 (15.3) | 93.5 (13.2) | 0.63 |
| Frailty index (frailty) | 3.3 (2.1) | 2.9 (1.6) | 0.29 |
| Mini-Cog (cognition) | 4.1 (1.4) | 4.5 (0.8) | 0.45 |
| SPPB-stance (physical function) | 3.9 (0.2) | 3.6 (0.5) | 0.09 |
| SPPB-lower limb strength (physical function) | 1.9 (0.3) | 1.8 (0.5) | 0.89 |

Supplementary table S2. Comparing the body composition and laboratory tests between completers and remainders.

| Variables | Completers | Remainders | *P* |
|---|---|---|---|
| Weight (kg) | 55.0 (12.3) | 57.4 (8.7) | 0.63 |
| Waist circumference (cm) | 77.5 (24.4) | 81.9 (9.3) | 0.74 |
| BMI (kg/m$^2$) | 24.2 (4.9) | 24.6 (3.9) | 0.87 |
| Calf circumference (cm) | 30.8 (9.5) | 33.8 (2.3) | 0.67 |
| Systolic blood pressure (mmHg) | 131.9 (40.7) | 133.0 (16.6) | 0.21 |
| Diastolic blood pressure (mmHg) | 72.1 (23.4) | 76.5 (9.3) | 0.80 |
| Fasting glucose (mg/dl) | 108.6 (16.0) | 102.3 (13.6) | 0.35 |
| Insulin (IU/L) | 7.4 (5.4) | 9.2 (7.4) | 0.86 |
| HOMA-IR (mg/IU) | 1.9 (1.4) | 2.4 (2.0) | 0.71 |
| Hemoglobin (mg/dl) | 12.9 (1.4) | 12.7 (1.8) | 0.75 |
| Triglyceride (mg/dl) | 108.4 (65.3) | 105.8 (60.3) | 1.00 |
| Cholesterol, total (mg/dl) | 175.9 (45.1) | 185.5 (52.5) | 0.65 |
| HDL-C (mg/dl) | 61.0 (17.7) | 58.4 (17.7) | 0.74 |

| | | | |
|---|---|---|---|
| LDL-C (mg/dl) | 93.2 (40.3) | 106.0 (57.4) | 0.70 |
| Creatinine (mg/dl) | 0.9 (0.4) | 0.9 (0.5) | 0.75 |
| AST | 23.0 (4.7) | 54.0 (89.1) | 0.89 |
| ALT | 16.7 (9.7) | 16.8 (5.2) | 0.41 |
| Limb BMC (g) | 721.2 (187.4) | 777.1 (326.9) | 0.61 |
| Limb fat (g) | 8458.9 (3507.0) | 10355.6 (2692.2) | 0.20 |
| Limb lean mass (g) | 12628.4 (2584.0) | 11332.8 (2190.1) | 0.24 |
| Muscle mass (kg) | 19.9 (3.9) | 21.1 (3.1) | 0.46 |
| Fat mass (kg) | 19.5 (9.3) | 19.8 (5.5) | 0.94 |
| Body fat ratio (%) | 34.2 (11.9) | 34.1 (5.7) | 0.98 |
| Waist-hip ratio | 0.9 (0.1) | 0.9 (0.1) | 0.32 |
| Abdominal fat ($cm^2$) | 153.1 (90.0) | 104.0 (62.3) | 0.18 |
| Subcutaneous fat (kg) | 16.6 (7.6) | 17.2 (4.4) | 0.83 |
| Basal metabolic rate(kcal) | 1065.2 (314.6) | 1182.3 (112.9) | 0.39 |
| Daily required energy (kcal) | 1748.7 (233.1) | 1820.3 (173.8) | 0.46 |
